# The Impact of Social Disparity on Prefrontal Function in Childhood

**DOI:** 10.1371/journal.pone.0035744

**Published:** 2012-04-26

**Authors:** Margaret A. Sheridan, Khaled Sarsour, Douglas Jutte, Mark D'Esposito, W. Thomas Boyce

**Affiliations:** 1 School of Public Health, Harvard University, Boston, Massachusetts, United States of America; 2 Children's Hospital Boston, Boston, Massachusetts, United States of America; 3 Eli Lilly and Company, Indianapolis, Indiana, United States of America; 4 School of Public Health, University of California, Berkeley, California, United States of America; 5 Helen Wills Neuroscience Institute, Berkeley, California, United States of America; 6 Psychology Department, University of California, Berkeley, California, United States of America; 7 College for Interdisciplinary Studies and Faculty of Medicine, University of British Columbia, Vancouver, British Columbia, Canada; University of Maryland, College Park, United States of America

## Abstract

The prefrontal cortex (PFC) develops from birth through late adolescence. This extended developmental trajectory provides many opportunities for experience to shape the structure and function of the PFC. To date, a few studies have reported links between parental socioeconomic status (SES) and prefrontal function in childhood, raising the possibility that aspects of environment associated with SES impact prefrontal function. Considering that behavioral measures of prefrontal function are associated with learning across multiple domains, this is an important area of investigation. In this study, we used fMRI to replicate previous findings, demonstrating an association between parental SES and PFC function during childhood. In addition, we present two hypothetical mechanisms by which SES could come to affect PFC function of this association: language environment and stress reactivity. We measured language use in the home environment and change in salivary cortisol before and after fMRI scanning. Complexity of family language, but not the child's own language use, was associated with both parental SES and PFC activation. Change in salivary cortisol was also associated with both SES and PFC activation. These observed associations emphasize the importance of both enrichment and adversity-reduction interventions in creating good developmental environments for all children.

## Introduction

It is well known that experience plays a central role in brain development. One example of this is the role that light exposure plays in columnar organization of the primary visual cortex [1]. However, at this time, it is not well understood what kinds of experiences are important in the development of higher-order association cortex or how fundamental aspects of plasticity play out in humans. The prefrontal cortex (PFC), one higher-order area, shows change in grey-matter volume from birth through late adolescence [2]. This extended developmental trajectory may provide opportunities for experience to shape the function of the PFC. However, to date, few studies have identified which aspects of experience are most likely to shape this cortical region. Because behavioral measures of prefrontal function are associated with learning and educational achievement across multiple domains [3], this is an important gap in our understanding of neural development. In the current study, we examined the association between one variable, parental socioeconomic status (SES), which is a marker for differences in environmental exposure, and prefrontal cortex function in children in middle childhood.

### Socioeconomic Status

SES is an aggregate measure intended to capture social standing, which is often estimated by identifying an individual's income, educational attainment, and job status. SES, measured in adulthood, is reliably associated with health outcomes [4]. In childhood, family SES can be estimated by measuring these variables with parent reports of household income and education. Low parental SES is associated with a higher incidence of risky health behaviors and lower academic performance for the child [5], [6], [7], [8], [9], [10]. Inequalities in health and academic achievement are evident early in childhood and persist or worsen across childhood and into adulthood [11]. It has been hypothesized that childhood inequality may shift the development of executive function leading to an increase in risky health behaviors in adulthood. In an example of the link between childhood inequality and adult health behaviors, Fujiwara and Kawachi (2009) demonstrated that failure to graduate from college was associated with smoking in high school [12]. This finding demonstrates that a third variable, such as exposure to inequality in childhood, is affecting both smoking behavior and college graduation rates.

The association of parental SES with broad aspects of childhood experience and with multiple important health and achievement indicators in adulthood has driven researchers to attempt to identify mechanisms by which social experience in childhood could shift developmental trajectories. The hypothesis that social experiences “get under the skin,” affecting child health through a variety of biological mediators, has been termed “biological embedding” [13]. Some accounts for observed linkages between childhood SES and health have focused on structural or material exposures, such as nutrition and health care. Such variables do not explain the broad association of parental SES with health, health behaviors, and achievement, nor do they account for the graded relation between SES and health outcomes, which exist even in the context of adequate health care and nutrition [4].

### The Prefrontal Cortex

The ability to hold in mind, and choose, future goals over current desires appears to be dependent, in part, on a set of cognitive functions termed executive function. Executive functioning is associated with a particular neural substrate, the prefrontal cortex (PFC), and is comprised of three cognitive abilities: working memory (the ability to hold relevant information and goals in mind), inhibition (the ability not to act on current desires or impulses, in the service of future goals), and switching (the ability to flexibly update goals or relevant information)[14]. Executive functioning is associated with better performance in school [15] and fewer negative health behaviors [16]. The PFC is necessary for the performance of simple working memory and inhibition tasks [17], [18], and PFC circuitry supports tasks that require multiple processes, such as planning, problem solving, and the learning of complex associations between stimuli and responses [19], [20]. This learning of complex associations between stimuli and responses, termed stimulus-response (SR) learning, has been observed to show a clear developmental progression across childhood, where SR learning improves with age [21] and prefrontal cortical development.

It is well established that the PFC has a long developmental trajectory, extending into late adolescence, with gross changes in volume and connectivity beginning early in childhood and continuing through early adulthood [22], [23], [24], [25]. Like all areas of cortex, the PFC shows a developmental inflection point in grey matter volume. Before this inflection point, which occurs in early adolescence, there is increasing grey matter volume. After this inflection point, there is steadily decreasing grey matter until early adulthood [26]. This loss in volume is thought to be the result of synaptic pruning and is a marker of cortical maturity. One of the last areas of PFC to mature is the dorsolateral prefrontal cortex, an area located in the middle frontal gyrus (MFG) and associated in many studies with the ability to hold in mind goals, plan complex behavior, and perform difficult tasks of working memory [18], [27], [28]. The principle of developmental plasticity—that areas of the brain in states of flux are most susceptible to the environments to which they are exposed—supports the idea that the extended developmental trajectory in the PFC, particularly in the MFG, provides multiple opportunities for SES-related environmental exposures to guide neural development [29]. Identifying which aspects of the environment are most associated with changes in prefrontal function is central to understanding the development of this region of the brain.

### SES and the Prefrontal Cortex

SES is a broad index of a family's social standing. SES in childhood is associated with physical and mental health, health behaviors, and achievement in childhood and adulthood. As early as kindergarten, parental SES is associated with performance on tests of executive function [30], [31], [32] [33], [34]. This association holds across countries and schooling environments [35], and these functional differences persist into adulthood. In one prospective study, adults' performance on a working memory task was strongly and significantly associated with their parent's SES [36]. In addition, there is accumulating neuroimaging evidence for SES-related differences in PFC function. In several studies using event-related potentials (ERP), children from lower SES families showed patterns of ERP components consistent with deficits in directed attention and inhibition [37], [38], [39]. However, none of these studies could precisely localize the differences in neural function to the prefrontal cortex because of the limitations of ERP technology.

### Mechanisms of the impact of SES

Two hypotheses have emerged from the field of epidemiology to account for the health and achievement effects of childhood SES: (a) SES effects are accounted for by differences in exposure to language [40] and more recently, (b) SES effects are accounted for by differences in exposure to stress and adversity [41]. These two theories are linked to two distinctive theories of intervention: (a) children from low SES families require increasingly enriched environments, including increased exposure to better and more complicated learning environments, and (b) children from low SES families require increased protection from the adversities that are more common in low SES neighborhoods and schools.

Currently, evidence exists for both hypotheses. Children from low SES environments are exposed to a decreased volume and complexity of home language use [42], [40] and they build vocabularies at a slower pace than children from higher SES families. Such differences are detectable during naturalistic observations, when vocabulary is formally tested, and across a variety of ages [43], [44]. Associations between SES and child language use are mediated by parental language use [44], indicating that these differences in language use are the result of differences in experience. It has been hypothesized that these socioeconomic disparities in childhood language development may account for differences in performance on many other tasks, including those involving executive function [30]. In such an account, it is children's relative abilities to use linguistic strategies in the problem-solving challenges of executive functioning tasks that lead to SES differences in performance on these tasks. An alternative explanation is that parental language use directly shapes childhood cognition by providing an opportunity to ‘practice’ certain cognitive operations, such as working memory [45]. For example, when more complex language structures are used, such as those involving conjunctions, holding in mind the first part of a sentence while listening for the meaning of the rest constitutes a working memory task. Increased exposure to this kind of language structure could plausibly constitute working memory practice, and thus lead to better performance on laboratory tests of executive function.

Low family SES is also associated with an increase in exposure to childhood adversity. These adversities range from exposures to violence, in the neighborhood and at home, to the level of disorganization in school environments [46], [47], [48]. Differences in executive function may be the downstream consequences of adversity-related exposures to stress over the course of childhood development. Exposures to adversity or stress in childhood are linked to physiological responses, which include activation of the hypothalamic-pituitary-adrenal (HPA) axis. Chronic activation of this regulatory system in response to stressors has a demonstrable impact on neural structures in human and animal studies. Studies in rodents have demonstrated, for example, that exposure to chronic stress results in decreases in dendritic spines in the prefrontal cortex [49]. In addition, multiple studies have linked baseline cortisol levels and SES in childhood [36], [41], [50], [51], [52]. Finally, one longitudinal study linked childhood SES to adult performance on a test of working memory and demonstrated that the association was mediated by childhood levels of allostatic load, a measure of the physiological impact of cumulative exposures to stressors [36].

In the current study, we first examined the hypothesis that the association between SES and prefrontal function is evident between 8 and 12 years in the context of a task with no explicit language demands [20]. Previously, with the use of this task in adults, recruitment of the PFC, specifically the MFG, was associated with successful task performance. We expected there to be significant associations between SES in childhood and activation of the PFC based on: (1) prior research linking SES and prefrontal function, (2) research demonstrating SES differences in behaviors that are dependent on the PFC, and (3) the principle of developmental plasticity. We measured the association between family SES and PFC function using functional magnetic resonance imaging (fMRI) in children performing a difficult SR learning task. Next, we examined several mechanisms by which this association might arise: (a) language exposure in the home, (b) child language use, and (c) child stress hormone exposure.

While our sample size was not large enough in this study to test a statistical mediation model, we were able to test linkages that lend support to the idea that a mediation model might be significant in a larger population of children. First, we propose that environments associated with low family SES result in increased exposure to chronic stress and therefore HPA axis activation. This increased exposure to stress hormones impacts PFC development and HPA axis reactivity to novel and challenging situations. Second, we posit that SES differences in environment exert influence through their effects on the development of language in children. Differences in the child's linguistic ability then shape other outcomes, including their performance on tests of executive function and associated prefrontal function [30], [53]. Lastly, we posit a direct effect of the linguistic environment on the development of the prefrontal cortex. In this model, advanced by Hart & Risley, (1995), the SES-related language environment in the home directly influences health and achievement behavior in children from families of differing SES.

## Methods

### Participants

**Figure 1:**
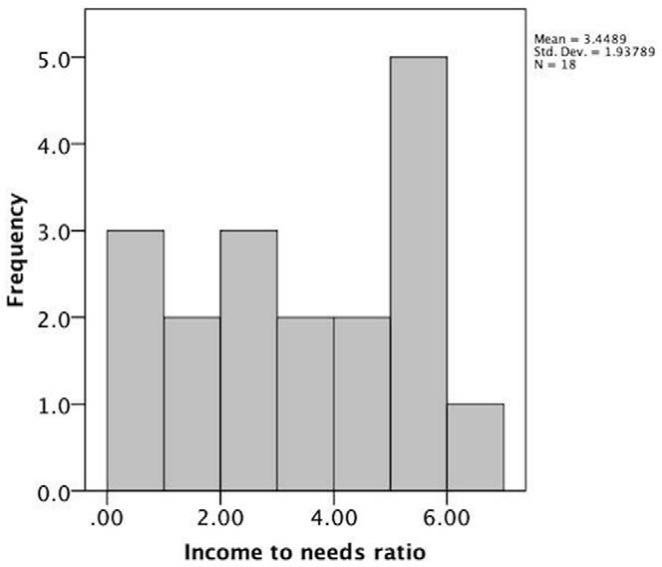
Distribution of income to needs ratio for the parents of children included in this study.

Participants were 20 children, of which 18 were scanned and two were excluded due to claustrophobia in the fMRI magnet. Of the 18 children who were scanned, 9 were from lower SES families (LSES) and 9 from higher SES families (HSES); ages ranged from 8–12 years (see Table 1 for characteristics of the sample). One high SES child was a boy, while all other children were girls. The analyses were completed with and without this child, and the results were unchanged. All children are thus included here, but the results of this study may only be generalizable to girls. The two excluded children were from LSES families. All participants were part of a larger group of subjects within the San Francisco Bay Area who participated in cognitive, behavioral, and home environmental assessments as part of a study of social determinants of neurodevelopment in middle childhood [54], [55]. Family SES in the present study was defined by a median split on an income-to-needs ratio, calculated by dividing the income of the participating family by the national poverty level income for a family of the same size (HSES ratio M = 5.1 SD = .79, LSES ratio M = 1.79 SD = 1.1, *t* = 7.37, p<.001). In Figure 1 we show the distribution of income-to-needs ratio, supporting our use of a median split. HSES and LSES groups were also significantly different in the primary caregiver's years of education (*t* = 3.14, p = .007), subjective socioeconomic status (*t* = 2.65, p = .02), relative to the country as a whole, using the MacArthur Ladder, [56] and wealth (*t* = 5.06, p<.001), as measured by asking parents how much money they would have if they subtracted their debt from savings and income. Behavioral and brain analyses were performed using direct statistical comparisons of the two groups and multiple regression when income-to-needs ratio was used as a continuous variable. All research was conducted with the approval of the Committee for the Protection of Human Subjects at the University of California, Berkeley.

**Table 1:** Characteristics of high and low socioeconomic status families in this study.

	High SES (n = 9)		Low SES (n = 9)	
Age at MRI, mean (SD), years	9.89	(1.05)	9.84	(1.09)
Parental Education (SD), years	17.2	(1.48)	14.3	(2.35)
Parental Wealth (SD), dollars (approximate)	91,875	(44,234)	10,500	(10,392)
Parental self report of status: USA (SD), scale 0–10	7.1	(1.6)	4.3	(2.7)
Parental self report of status: Community (SD), scale 0–10	6.8	(1.2)	6.3	(2.6)
Mom Reported Stress (SD), Total score (scale 0–50)	24.8	(4.98)	27.2	(11.02)

### Behavioral Task

Child participants learned to perform a stimulus-response (SR) mapping task. Such tasks are frequently used with children to study the development of executive function (i.e., dimensional change card sorting task (DCCS)[21]. In DCCS studies, subjects learn to associate one response with one family of stimuli (e.g., blue stimuli) and another response with another family of stimuli (e.g., red stimuli). In the current study, participants learned to associate one out of four possible button presses with one family of stimuli and another button press with another family of stimuli. One such set of associations (e.g., button 1 with family A and button 4 with family B) is called a rule. Unlike the traditional forms of SR mapping tasks, in this rule, distinguishing one class of stimuli from another was difficult and required the child to recognize and use a visually complex pattern. The increased complexity of stimuli in this task elicits sustained PFC activity during learning in adults [20]. Given the lack of clear verbal labels in this task design, we reasoned that SES differences on a complex executive functioning task could be assessed while making minimal direct demands on verbal processing. The task, thus, allowed us to separate the indirect effects of language environment on neural development from the direct effects of language ability on task performance.

**Figure 2:**
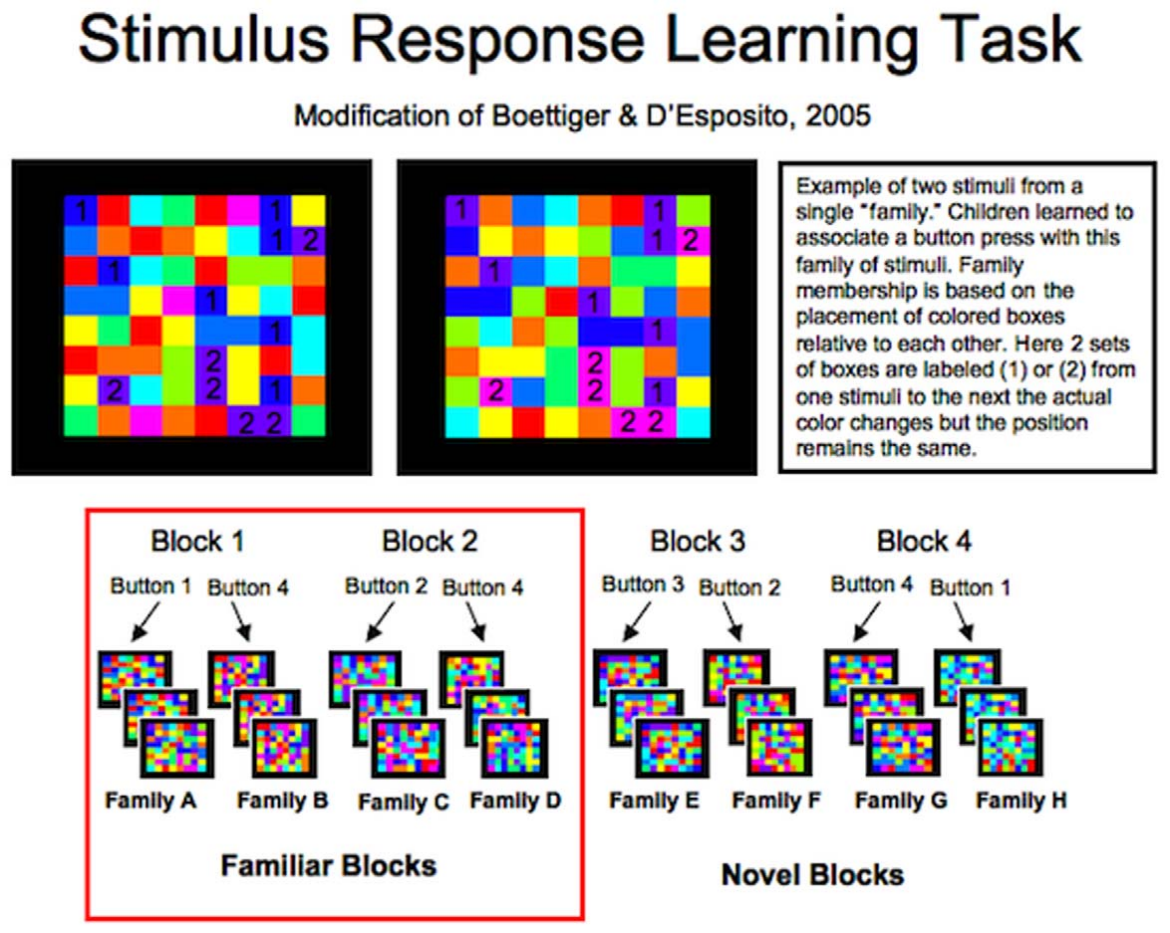
Example of stimuli used in task. (*Top*) Two stimuli from the same family: although the colors change from one exemplar of a family category to another, the pattern of colors remain the same. In the left exemplar, the “1” blocks are blue, but in the right exemplar, these same blocks are purple. Similarly, the blocks which are purple in the left exemplar are “2” blocks, but these same blocks are pink in the right exemplar. (*Bottom*) Examples of all the families of stimuli used in this task. During each block, participants were taught to distinguish between 2 families of stimuli. They were taught to press one button when shown exemplars from one family and another button when shown exemplars of the other families. All together 4 possible button presses were used during the course of the task (1, 2, 3, or 4); chance performance was 25%.

**Figure 3:**
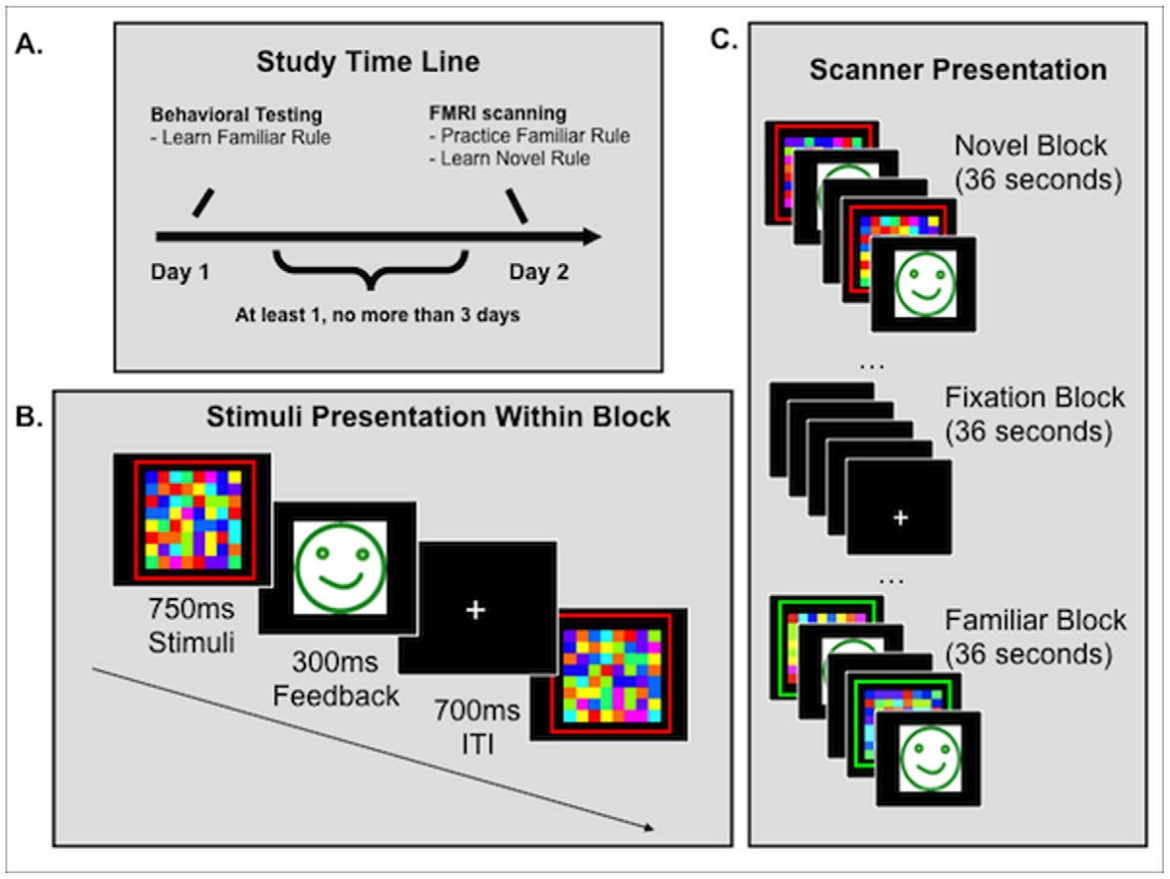
Depiction of study timing across days. **A.** ***Time line of study.*** All subjects participated in a behavioral session before the fMRI session where they learned two rules to criterion (80% accuracy). In each rule (presented as 1 block), they distinguished with a button press between 2 families of stimuli. These rules are designated Familiar Rules. During the fMRI session, they practiced these rules on some blocks and on other blocks learned 2 new rules, designated Novel Rules. **B.** ***Task presentation during behavioral training and fMRI scanning***. Each exemplar of a family was presented for 750 ms, during which time participants responded with a button press indicating which family it belonged to. Their response was followed by feedback indicating if this response was correct or not. Feedback was either a green smiley face or a red frowny face. Finally, this was followed by a 700 ms intertrial interval (ITI) **C.** ***Scanner Presentation.*** Stimuli were presented in a blocked design. Outline (here in red or green) indicates the kind of rule being performed (Familiar 1 or 2 or Novel 1 or 2).

During an initial session, each child learned two SR mapping rules (Familiar Rules) to a criterion of 80% accuracy (see Figure 2 for examples of the stimuli). Criteria were set at achieving a level of accuracy, instead of the number of training trials, to ensure that participants from both high and low SES families began the fMRI task with the same level of task proficiency and to maintain consistency with other published versions of this task [20]. During training, children were provided with as much guidance as needed to learn the Familiar Rules, and any questions children had about the task were answered by an experimenter who worked individually with the child. During this session each child also underwent a ‘mock scan.’ This session served to accustom children to the scanning environment and reduce their anxiety around scanning (see Figure 3).

**Figure 4:**
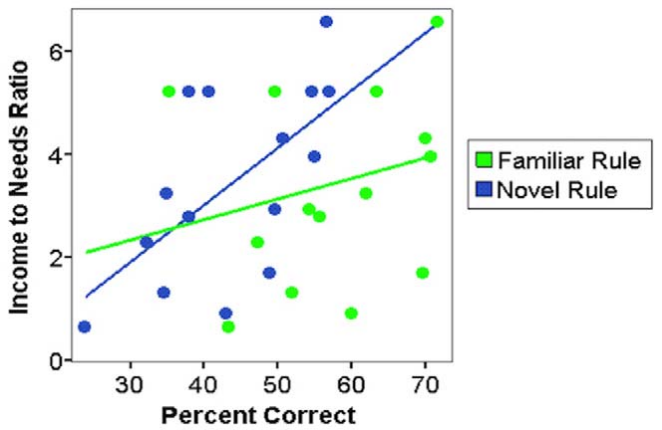
The association between income-to-needs ratio and accuracy on the behavioral task. A significant association exists for novel rule accuracy (blue), whereas for familiar rule accuracy (green) the association is non-significant, which is consistent with the fact that both groups were trained to 80% accuracy prior to scanning.

Between one and three days after training, subjects returned for an fMRI session, during which they practiced the Familiar Rules and learned two new rules (Novel Rules). Overall accuracy on this task was low: average Familiar Rule accuracy was 58% (SD = 10.1%), and average Novel Rule accuracy was 44% (SD = 10%). With four possible responses from which to choose, chance performance was 25% (see Figure 4). When analyzing fMRI data, activation to these two tasks are contrasted with each other: Novel rule activity > Familiar rule activity, allowing us to isolate activity to learning without examining unrelated to aspects of the stimuli (e.g., color, shape). This contrast is referred to as ‘activity during learning’; it is also referred to as BOLD activation to learning or BOLD signal associated with learning within the text.

### FMRI Methods

Functional magnetic resonance imaging (fMRI) was used to acquire blood oxygen level dependent (BOLD) signal using a 4.0 T Varian INOVA MR Scanner with standard scanning procedures. Each subject viewed four runs of the SR mapping task, and during each run she/he was exposed to five Novel and five Familiar Rule blocks. Stimuli were presented in blocks of 15 trials (e.g., Familiar Rule A would be presented for 15 trials, then Novel Rule B, Familiar Rule B, Novel Rule A). Blocks were presented in a fixed order to all subjects. In total, the task consisted of 20 blocks of Familiar Rules (10 of Familiar A and 10 of Familiar B) and 20 blocks of Novel Rules (10 of Novel A and 10 of Novel B) for a total of 300 stimuli of each type. Because there were no significant predicted or actual differences in task performance or neural activity within stimuli type (e.g. between Familiar A and B), all analyses are collapsed across this dimension.

Functional volumes were acquired in the coronal plane using 20 slices of 3.5 mm isomorphic voxels to facilitate signal acquisition in the PFC. These parameters allowed for coverage of the area from approximately the posterior precentral sulcus continuing anteriorally to cover the entire frontal lobe and portions of the temporal lobe and associated sub-cortical structures. Motion correction was accomplished using a 6-parameter rigid-body transformation [57]. Prior to individual analysis, data were normalized to Montreal Neurological Institute (MNI) space. Any single acquisition where the subject moved 3 mm from the first acquisition or 1 mm from the preceding acquisition was removed from the analysis using an outlier covariate (http://www.nitrc.org/projects/artifact_detect/). No subjects had to be excluded due to excess movement. Image processing and analysis were completed using statistical parametric mapping (SPM2; http://www.fil.ion.ucl.ac.uk/spm/software/spm2/) and linear combinations of the covariates modeling each condition.

The results of the individual analyses were combined into a group analysis to identify differences in BOLD response by rule type (Familiar, Novel) and SES group (HSES, LSES). This experiment was constructed as a cognitive subtraction, where aspects of stimuli presentation were held constant across Familiar and Novel blocks, but the state of rule acquisition was different. Thus, all reported analyses are for the direct contrast of Novel compared to Familiar Rules, highlighting activity due to learning. BOLD signal for children from HSES and LSES families was examined separately using one sample t-tests. In addition, BOLD signal for children from LSES families was directly compared to children from HSES families using two-sample t-tests. All presented findings were significant at p<.05 or p<.001, cluster-level corrected for multiple comparisons using fmristat unless stated otherwise [58]; see also http://www.math.mcgill.ca/keith/fmristat).

### Salivary Cortisol Methods

To test the associations between SES, prefrontal function, and HPA axis activation, salivary cortisol level was measured in children pre- and post-fMRI scanning. Approximately 30 minutes before fMRI scanning, children were asked to chew on a sterile Salivette for one minute. Five to ten minutes after scanning and approximately one hour after saliva sample 1 was collected, a second sample was collected. Salivettes were stored in a freezer at −20°C and were analyzed at the laboratory of Biopsychology, TU Dresden, Germany. Salivary cortisol concentrations were measured using a commercial immunoassay with chemiluminescence detection (CLIA; IBL Hamburg, Germany). Once results of this analysis were returned, the percent change in cortisol 2 compared to cortisol 1 was calculated using the formula (cortisol 2 – cortisol 1)/cortisol 1)*100. Percent change in cortisol was used instead of a direct change score because it was hypothesized that the size of the change could be related to the starting cortisol value [59] and previous research has associated initial cortisol with SES in children [52].

Two subjects in this sample did not have saliva samples collected due to procedural errors. Of the 16 subjects with saliva samples, 13 participated in fMRI scanning in the late afternoon, and 3 were assayed during the morning hours. For all analyses using cortisol measures, the time of day that subjects were assayed was entered as a control variable. Normatively at these times (morning and afternoon), salivary cortisol would decrease across the hour of scanning due to the circadian decline in basal cortisol secretion [60]. Thus, an increase in cortisol across the time of the scan would indicate a stress response and a diversion from this circadian rhythm; no change in cortisol before and after scanning would indicate a slight activation of the HPA axis and a diversion from the circadian rhythm; and a decrease in cortisol across the time of the scan would indicate no HPA axis activation to the scanning environment, consistent with the circadian rhythm.

### Child and Family Language Methods

Each study family participated in a videotaped, unstructured family dinner (following the methods of Hart & Risley, 1995). Dinner was chosen by the family and purchased by the researchers, then delivered to the family's home. The family was told, as part of the larger study, that we were interested in all aspects of family interaction and they should behave normally and have dinner as they usually would. This conversation was transcribed and several dimensions of language use were calculated using Systematic Analysis of Language Transcripts (SALT) software (Language Analysis Lab, 2006). SALT software automatically classifies words and parts of words using a reference database developed with children in our age range from California and Wisconsin. Language use was coded separately for the target child and other family members. Language complexity was calculated as the sum of word roots, bound morphemes, and conjunctions that were uttered during mealtime conversation (30 minutes). These represented vocabulary complexity, word complexity and sentence complexity, respectively. These measures of spoken language by family members, not including the target child, were used to operationalize Family Language Complexity, while these measures of spoken language by the target child was used to operationalize Child Language Complexity. Because language complexity could increase as a function of the total amount of language used in a conversation, we calculated two control variables: family and child words per minute (WPM). These variables took the exact number of minutes for which the family had dinner and divided it by the total number of words uttered by (a) any member of the family (not including the target child: family WPM) or (b) the target child (child WPM). This variable was included as a control when estimating the effect of family or child language complexity on outcomes. Family WPM was non-significantly associated with family language complexity (*r* (18) = .31, p = *n.s.*), while child WPM was significantly associated with child language complexity (*r* (18) = .66, p = .003). Child and family WPM were non-significantly and negatively correlated with each other (*r* (18) = −.25, p = *n.s.*). Finally, child and family language complexity were non-significantly and positively correlated with each other (*r* (18) = .31, p = *n.s.*).

### Statistical Analysis

SES was treated as both a dichotomous (median split of income-to-needs ratio) and a continuous variable, as appropriate to the particular analysis. The dichotomous analysis allowed for the use of a two-sample t-test to identify activation in the prefrontal cortex that differed between children from high and low SES families when children were in the context of learning (i.e., a direct contrast of familiar and novel rules). For behavioral data, SES and rule familiarity were entered into a 2×2 mixed ANOVA to examine the effect of SES on accuracy to the familiar and unfamiliar rule performed at the scanner.

To determine the significance of relations between continuous predictors (e.g., age, income-to-needs ratio) and dependent measures (e.g., task accuracy), as well as statistical mediation by family and individual difference variables, ordinary least-squares (OLS) multiple regression was used. The models included a basic model that examined income-to-needs ratio, child age, and time of day scanned as predictors of Novel Rule accuracy. In addition, three other models were employed. These models considered the following predictors: percent change in salivary cortisol across the time of scanning, child language complexity during a dinner conversation, and family language complexity during a dinner conversation. We tested each of these three predictors of income-to-needs including controls for child age and time of day scanned. For the language variables we included controls for family words per minute (WPM) or child words per minute (WPM). Finally, these four models were then tested as predictors of task-associated activity in the right MFG. Because right MFG activity was identified using a contrast (HSES vs. LSES) defined in part by income-to-needs ratio, the association between income-to-needs and MFG activity is to be expected, however the use of these models allowed us to examine the linear associations between these variables and task related neural activity in addition to testing the dichotomous association between these variables.

## Results

### Behavioral Performance

**Figure 5:**
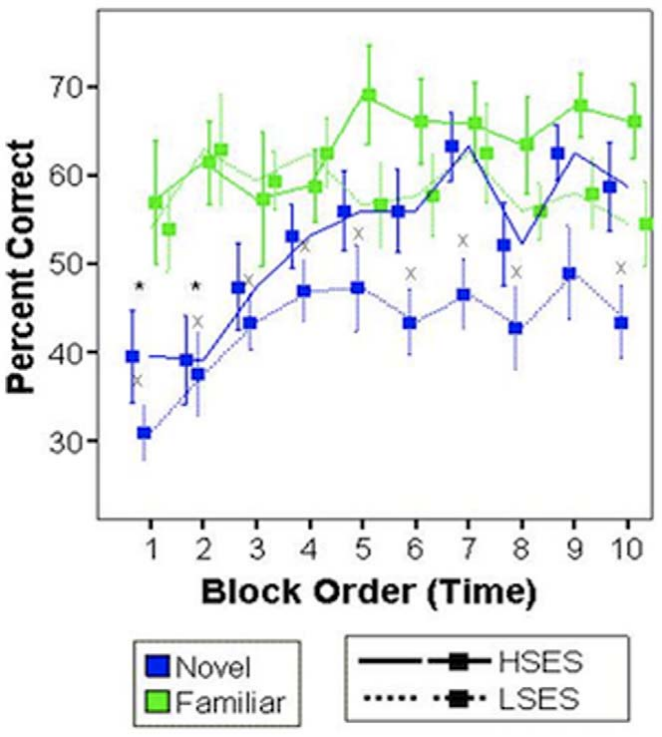
Learning curves for children from high and low SES families for accuracy performance on both Novel (blue) and Familiar (green) rules. Data at each time point is collapsed across instances of Familiar (A/B) and instances of Novel (A/B) rules, yielding 10 time points from an original 20 if instances of rules were viewed separately. HSES children performed significantly less accurately on Novel compared to Familiar rules during early blocks of the scanner task (asterisks) whereas LSES children performed more poorly on the Novel compared to Familiar rule throughout the scan. (*) indicates a significant difference for Familiar Rule Accuracy > Novel Rule Accuracy for HSES participants (solid lines); (x) indicates a significant Familiar Rule Accuracy > Novel Rule Accuracy for LSES participants (dotted lines).

A mixed (within X between) 2×2 ANOVA (rule familiarity X SES) revealed an expected main effect of rule familiarity on accuracy (*F* = 35.7, *p*<.001) and a main effect of SES on accuracy (*F* = 4.44, *p* = .05). Both rule familiarity and high SES improved performance. Next, to assess the continuous association between income-to-needs ratio and accuracy, income-to-needs ratio, age, and the time of day the subject was scanned were entered into a multiple regression equation predicting accuracy. Income-to-needs ratio was a significant, independent predictor of Novel (*t* = 5.54, *p* = .015), but not Familiar Rule accuracy (*t* = .424, *p* = .74; Figure 4) in the expected direction: increased income-to-needs resulted in improved performance. This finding is consistent with the fact that both groups were trained to criterion on the Familiar Rule prior to scanning. Learning curves were plotted for each of the groups and rule types separately, and performance at each time point was compared within subjects to examine the difference between novel and familiar rules at each time point. A decrease in significance between performance on novel and familiar rules at block 4 compared with block 3 would indicate learning, as performance on novel rules approached performance on already learned familiar rules. This analysis revealed that both HSES and LSES children improved in their performance on the novel SR mappings during the first 5 blocks. However, in the second half of the experiment, HSES children continued to improve, and LSES children did not (Figure 5). To further quantify this learning difference, the first and last 5 blocks were considered separately. When the first 5 blocks were considered alone, both HSES and LSES participants performed with lower accuracy on Novel Rule blocks compared to Familiar Rule blocks (Novel vs. Familiar Rules early trials HSES *t* = 2.68, p = .055; LSES t = 7.51, p = .002). However, during the last five blocks of rule learning, only LSES children continued to perform poorly on the Novel Rule blocks (Novel vs. Familiar Rules late trials HSES *t* = .16, p = .884; LSES t = 6.54, p = .003). The mean accuracy on Familiar Rule trials for the first five blocks was LSES = 59% and HSES = 61%; for the last five blocks, LSES = 58% and HSES = 66%. The mean accuracy on Novel Rule trials for the first five blocks was LSES = 41% and HSES = 47%; for the last five blocks it was LSES = 45% and HSES = 59%. Thus, at no time was accuracy lower than chance (25%), but task difficulty was high, even for familiar rules.

### FMRI data

**Figure 6:**
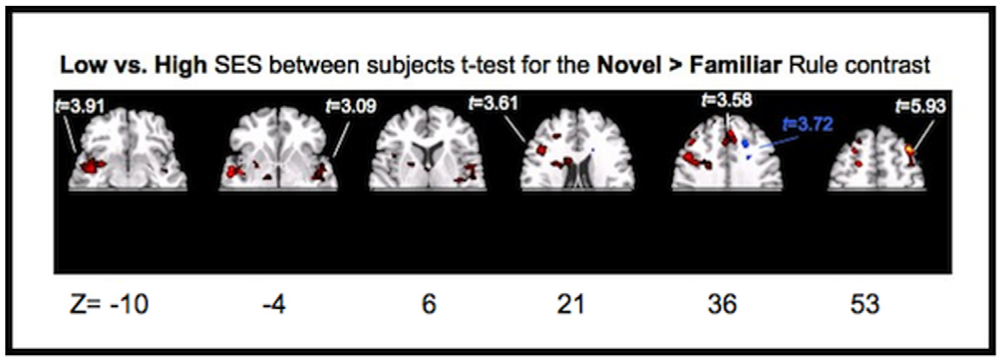
Significantly active areas (p<.05, cluster level corrected) for the two sample t-test comparing children from low socioeconomic status families to children from high socioeconomic status families for the novel rule > familiar rule contrast. Activation in red represents areas that were more active for children from low socioeconomic status families during learning. Activation in blue represents areas more active for children from high socioeconomic status families during learning. Circled in red is the RMFG, which survived further correction at p<.001 cluster level correction.

To determine how children recruited neural structures in the service of learning, activation to practicing the Familiar Rules was directly contrasted with activation to learning Novel Rules within each subject. When children from LSES families were considered alone, they showed increased activation for Novel relative to Familiar rules in a variety of cortical (right and left middle frontal gyrus) and sub-cortical (hippocampus, midbrain) areas (Table 2). Children from HSES families showed increased activation only in the left superior frontal gyrus (Table 2). When brain activity was compared between children from LSES and HSES families using a two sample t-test, there was significantly more activity for LSES participants in areas previously associated with rule learning, including the supplementary motor area, basal ganglia, bilateral inferior frontal gyrus, anterior cingulate cortex, and right middle frontal gyrus (RMFG) (Table 2). Only one area, in right superior frontal sulcus, was more active for HSES compared to LSES children during learning. All reported results were significant at p<.05, cluster level corrected (with a 296 voxel extent; Figure 6). Of these activations, only increased activation of the RMFG for LSES compared to HSES was still significantly active at p<.001, cluster level corrected (18 voxel extent; x = 26, y = 44, z = 44 *t* = 5.38; Figure 6). Thus, children from LSES families activated the RMFG more than their HSES peers in the context of poorer task performance. Increased neural recruitment during a task in the context of worse or equivalent performance may reflect compensatory recruitment of areas not usually employed in the process of learning. Alternatively, it may reflect that children from LSES families, who took longer to acquire the Novel Rules, are “in the learning context” longer than their peers from HSES families.

**Table 2:** Early Trials: Activity for learning with behavior equated.

Novel - Familiar (contrast)		
High only (t-test)		
t-values	coordinates	Area
4.97	−30 −10 60	L Superior Frontal Sulcus
**Low only (t-test)**		
4.54	26 48 44	R Middle Frontal Gyrus
4.80	−40 44 38	L Middle Frontal Gyrus
3.52	0 38 22	Anterior Cingulate Cortex
2.55	−20 −8 −20	L Hippocampus
5.53	54 −2 −16	R Middle Temporal Gyrus
3.92	−6 −12 −8	L Midbrain
**Low > High (2 sample t-test)**		
5.51	26 48 44	R Middle Frontal Gyrus
3.40	−44 44 32	L Middle Frontal Gyrus
4.32	0 38 22	Anterior Cingulate Cortex
2.84	−46 2 26	L Precentral Gyrus
3.54	−40 −10 8	L Posterior Insula
2.73	−10 8 18	L Caudate
3.05	−30 −14 −24	L Hippocampus
3.84	−2 −14 0	Mid Brain Nuclei
**High > Low (2 sample t-test)**		
3.24	20 −6 52	R Superior Frontal Sulcus

**All Trials: Activity for Learning**. (*Top*) Significantly active areas for children from either LSES or HSES families for novel rule > familiar rule contrast (one sample t-test). (*Bottom*) Significantly active areas for the two sample t-test comparing children from LSES families to children from HSES families for the novel rule > familiar rule contrast. Only areas that were above baseline for one group compared to the other are reported (e.g., no area that was below baseline for children from HSES families, but at baseline for LSES families was reported).

To determine if this increase in neural activity actually reflected a prolonged acquisition phase, we performed two further analyses. First, we examined activity to learning for the initial 5 blocks of the task when both HSES and LSES children were in the process of acquiring new rules. Second, we performed a region of interest (ROI) analysis to examine the association between learning and activation of the RMFG.

In the case of activity to learning during the first 5 blocks, we would expect that if RMFG activation is strictly an index of being in the ‘learning state’ across both groups, both HSES and LSES children would activate the RMFG in the context of learning. In addition, we would expect that HSES children would activate this area more strongly in the first 5 blocks when their learning was maximal and effective. Instead, we observed that during the first five blocks LSES children activate the RMFG in the context of learning and HSES children do not (Table 3). When learning, related activations for HSES children were directly compared to LSES children via 2-sample t-test: the only area that was significantly more active for HSES children was the left superior frontal sulcus. These findings are not conclusive, but are congruent with the hypothesis that the increase in RMFG activity for LSES children reflects a specific inefficiency in recruitment, instead of more time in the context of learning.

**Table 3:** Brain activity during early trials: Activity for learning with behavior equated.

Novel - Familiar *Early Trials Only* (contrast)		
High only (t-test)		
t-values	Coordinates	Area
7.36	−20 10 44	L Superior Frontal Sulcus
4.43	36 −2 50	R Superior Frontal Sulcus
4.24	−12 30 44	L Anterior Superior Frontal Sulcus
8.02	−6 −4 34	Middle Cingulate Gyrus
5.08	20 0 24	R Caudate
**Low only (t-test)**		
3.47	−18 44 24	L Middle Frontal Gyrus/Superior Frontal Sulcus
6.78	−12 14 44	L Superior Frontal Gyrus
3.51	26 52 44	R Middle Frontal Gyrus (sub-threshold, cluster size = 83)
6.63	6 0 36	R Middle Cingulate Cortex
3.35	−22 −8 4	L Putamen/Globus Pallidus
4.02	14 −22 −2	R/L Midbrain
**Low > High (2 sample t-test)**		
2.86	−22 −12 2	L Putamen/Globus Pallidus
3.41	−50 −4 −8	L Superior Temporal Sulcus
3.84	−6 −12 −8	L Midbrain
**High > Low (2 sample t-test)**		
4.89	−28 34 48	L Superior Frontal Sulcus/L Middle Frontal Gyrus

(*Top*) Significantly active areas for children from either LSES or HSES families for the early trials of the novel rule > familiar rule contrast (one sample t-test). (*Bottom*) Significantly active areas for the two sample t-test comparing children from LSES families to children from HSES families for the early trials of the novel rule > familiar rule contrast. Only areas that were above baseline for one group compared to the other are reported.

Next, we directly examined how and if increased activation of the RMFG supported performance on this task by performing a region of interest (ROI) analysis. To identify this ROI, BOLD activity related to learning (direct contrast of Novel > Familiar Rule), which was also significantly increased for LSES compared to HSES subjects (two sample t-test), was identified. BOLD activity in this functionally defined region was then extracted for each subject in each condition (Novel and Familiar rule). To isolate activity to learning, Novel and Familiar rule activity was directly contrasted within this ROI (Novel > Familiar). This ROI is hereafter referred to as the RMFG ROI.

We used OLS multiple regression to determine if this activity was significantly associated with Novel Rule accuracy after controlling for age and time of day scanned across both HSES and LSES children. There was an inverse association with Novel Rule accuracy that approached significance (*B* = −1.6, *t* (14) = −1.8 p = .09). When this activation was examined separately for HSES and LSES groups using correlation, activation was positively associated with performance in the LSES group (*r* (8) = .60, p<.08) and negatively associated with performance in the HSES group (*r* (8) = −.46, p<.21), although neither association was significant. In addition, activity related to learning in this ROI was highly and inversely associated with income-to-needs ratio after controlling for participant age, time of day scanned, and task performance (*B* = −.86, *t* (14) = −2.69 p = .02; see also Table 4, Model II). This is less surprising because this ROI was identified via a direct comparison of learning related activity between HSES and LSES children, however the direction of this association held up when examined separately for HSES and LSES children using bivariate correlation. The association between income-to-needs ratio was, in fact, strongest within the LSES group (*r* (8) = −.44, p = .24), likely due to the increased variance in income-to-needs within this group.

**Table 4:** Results from the regression equation, unstandardized Beta values are listed first and t-statistics are in parentheses; significance is indicated using either **, *, or +(** p<0.01, *p<0.05, +p<.10).

Regression Table for Language Variables		
Model I	(a) Novel Rule: Percent Correct	(b) Activity to Learning: BOLD
Age (years)	5.51 (2.89)**	.342 (.743)
Time into the Scanner (hours)	.207 (.366)	.029 (.044)
**Income-to-needs Ratio**	2.81 (2.69)**	−.897 (−3.57)**
*Constant*	*−22.85 (−.914)*	.*072 (.012)*

Model II		
Age (years)	3.92 (1.94)	.516 (1.62)
Time into the Scanner (hours)	.356 (.577)	−.112 (−1.15)
Parent/Family Words per minute	0.024 (.335)	−.015 (−1.33)
**Parent/Family Language Complexity**	.075 (2.43)*	−.016 (−3.32)**
*Constant*	*−13.18 (−.534)*	.*389 (.10)*

Model III		
Age (years)	4.25 (1.8)	.41 (.966)
Time into the Scanner (hours)	−.394 (−.532)	.116 (.875)
Child Words Per Minute	−.129 (−.405)	−.061 (−1.07)
Child Language Complexity	−.055 (−.492)	−0029 (−.145)
*Constant*	*13.5 (.457)*	*−4.194 (−.795)*

Model IV		
Age (years)	3.95(1.45)	.50(.833)
Time into the Scanner (hours)	−.220(−.311)	.07(.657)
**Percent Change in Cortisol**	**−.166 (−2.04)+**	.04(2.36)*
*Constant*	*18.13(.596)*	*−7.86(−1.16)*

Models I–IV show simple associations between SES, family language complexity or child language complexity (measured during a dinner time conversation), percent change in salivary cortisol (over the time of fMRI scanning) and (a) novel rule accuracy or (b) activation to in the right middle frontal gyrus region of interest. Model IV includes 15 children; 3 children were dropped from this analysis because their cortisol samples were not collected (2 subjects) or the values acquired for them were more than 3 standard deviations from the mean (1 subject).

### SES, Child Language, and Cortisol Reactivity

To determine the association between (a) task performance and (b) task associated neural activity in our RMFG ROI and our predictor variables (SES, child/family language, cortisol reactivity), we tested the significance of four basic models using OLS multiple regression, labeled Models I–IV (Table 4). In Models I (a) and (b), we demonstrated that after controlling for child age and time of day scanned, there was a significant association between income-to-needs ratio and (a) Novel Rule accuracy and (b) activation in the RMFG ROI during learning extracted for each subject (described above; see Table 4 for results). Next, in Model II (a) and (b), we identified significant associations between family language complexity and (a) Novel Rule accuracy and (b) activation in the RMFG ROI. Unlike associations with income-to-needs, which are expected given the definition of the RMFG ROI (by a median split on income-to-needs), this association is independent. Family language complexity was defined as the sum of word roots, bound morphemes, and conjunctions (see above). In this model, we introduce an additional control, family WPM, to account for different rates in language production across families. The final model included family WPM, child age, time of day scanned, and family language complexity. There were no significant associations between family WPM and our outcome variables (Table 4). In Model III, we did not observe a significant association between child language complexity and (a) Novel Rule accuracy or (b) activation in the RMFG ROI (Table 4). The final model included child WPM, child age, time of day scanned, and child language complexity. Finally, in Model IV, we demonstrated a significant association between percent change in cortisol over the time of scanning and activation in the RMFG ROI, but not (a) Novel Rule accuracy.

### SES and Potential Pathways

To better understand if percent change in salivary cortisol across the scan, complexity of the home language environment, and the child's own language complexity could serve as pathways by which SES came to affect prefrontal function and associated behavior, we used OLS regression. First, we tested a model including child age, time of day scanned, family WPM, and income-to-needs ratio as predictors and family language complexity as an outcome. In this model, the association between income-to-needs ratio and family language complexity was significantly positive (*B* = 26.96, *t* (17) = 4.25, *p* = .001); as income-to-needs ratio increased, family language complexity increased. Child age, time of day scanned, and family WPM were not significant predictors of family language complexity. Next, we tested a model including child age, time of day scanned, child WPM, and income-to-needs ratio as predictors and child language complexity as an outcome. In this model, the association between income-to-needs ratio and family language complexity was non-significant (*B* = 4.57, *t* (17) = 1.59, *p* = .13). Child age and time of day scanned were not significant predictors of child language complexity. However, child WPM was significantly and positively related to child language complexity (*B* = 2.13, *t* (17) = 4.48, *p* = .001). Finally, we tested a model including child age, time of day scanned, and income-to-needs ratio as predictors and percent change in salivary cortisol as an outcome. In this model, the association between income-to-needs ratio and change in salivary cortisol was significantly negative (*B* = −12.21, *t* (13) = −2.86, *p* = .017); as income-to-needs ratio increased, change in salivary cortisol decreased. No child in the study increased in cortisol over and above the baseline value, indicating that participating in a neuroimaging experiment did not serve as a stressor capable of activating the HPA axis. This is consistent with our expectations and efforts to make the experience as benign as possible for all participants. However, while this experience did not activate the HPA axis stress response, it was quite difficult and required substantial concentration over a sustained period. In response to this challenge, some children showed slight activation of the HPA axis consistent with the effort needed to meet this challenge, and a deviation from the diurnal rhythm. These children were overwhelmingly from high SES families, resulting in the positive association between percent change in salivary cortisol and SES observed here. Neither child age nor time of day scanned was a significant predictor of change in salivary cortisol.

## Discussion

**Figure 7:**
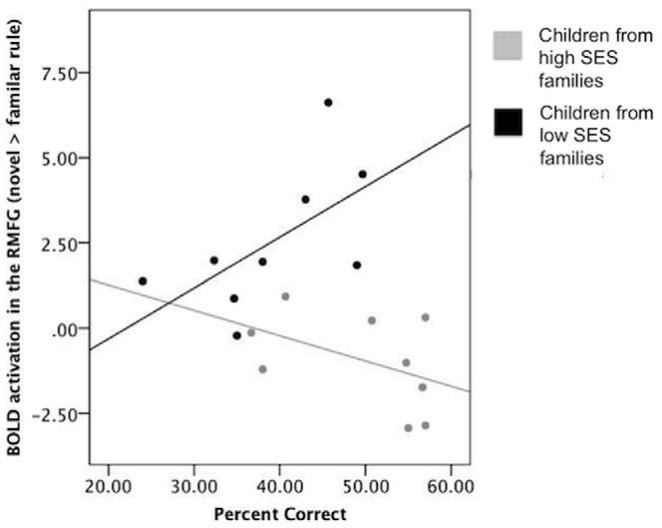
Non-significant associations between BOLD activity in the RMFG to learning and behavioral performance for HSES and LSES children separately.

We present data demonstrating a negative association between family SES and (a) errors in acquiring a novel stimulus response association and (b) activation of the right middle frontal gyrus (RMFG) in the context of learning for children aged 8–12 years. These results offer further confirmation of prior findings linking measures of family SES with child prefrontal functioning [32], [61], [62]. The area which best distinguishes HSES and LSES children is the RMFG, which is highly active early during learning of this task in adults, but which decreases in activation across rule acquisition [20]. Interestingly, we observed that activity in this area was positively correlated with performance for children in the LSES group and negatively correlated with performance for children in the HSES group (Figure 7). While these associations were non-significant, this mirrors the high RMFG activation during learning for adults and supports the idea that children from LSES families are spending more time learning the rule associations and this drives the greater recruitment of the RMFG observed in this group.

Additionally, we tested three potential pathways by which SES might come to be associated with prefrontal function, all of which have previously received partial experimental support: family language complexity, child language complexity, and change in the child's salivary cortisol. Of these potential pathways only salivary cortisol and family language complexity were associated with (a) task accuracy, (b) RMFG during learning, and (c) family SES.

Previous epidemiologic research has consistently demonstrated that children from lower SES families are at an increased risk for health and academic problems compared to their peers from high SES families [5]–[10]. Multiple theories for how social experiences associated with SES could get “under the skin” and affect health outcomes have been proposed. Among these, we tested three central theories that have been specifically linked with child cognitive and neural function. In one theory, the broad range of SES effects are accounted for by differences across SES in exposure to language in the home environment [40]. Findings from the study reported here provide support for this theory. We measured language use in the home environment separately for the target child and his/her family and demonstrated that family language complexity predicts both task performance and associated neural activation in the context of learning, even after controlling for the rate of language production in the family. In addition, family language complexity was significantly and positively associated with income-to-needs ratio. We take this as evidence that the association between income-to-needs ratio and our child performance variables may be indirect and through the pathway of language exposure in the home.

The second theory proposes another possible pathway: language exposure in the home could affect health and achievement only through its influence on child language function. This constitutes a second model by which SES could come to affect cognitive and neural function. This pathway has been proposed primarily in the context of measuring cognitive function in children, and thus, we considered it here. The theory proposes that exposure to language use in the home specifically shapes a child's language ability, and this, in turn, affects their cognitive performance on tasks in the laboratory or at school. In a previous study of kindergarteners examining behavioral performance on cognitive tests, differences in child language ability mediated the effect of SES on children's performance of tasks associated with prefrontal function [30]. In the current study, we do not find support for this theory. Child language complexity does not predict task performance on the stimulus-response learning task, nor does it predict task-related neural activity in the prefrontal cortex. In this sample, it appears that the effect of language exposure in the home is not an indirect effect of child language ability. Our results differ from previous results, potentially because of differences in age (8–12 years of age compared to kindergarten) and because of the kind of task we chose to use. In this study, we used a SR mapping task similar to those used in studies of cognitive development (e.g., Dimensional Change Card Sorting), but designed to increase utilization of non-verbal strategies by employing difficult to name stimuli and a rule that was not easily verbalized. Perhaps, if our stimuli were more easily named, child language complexity would mediate the association between income-to-needs ratio and task performance.

A third possible way in which SES could come to influence health and achievement concerns exposure to adversity and resulting changes in regulatory systems, such as the HPA axis in children from lower SES families. In rodents, exposure to stressful events early in life is associated with disruptions of the HPA axis and neuro-structural changes in the hippocampus and prefrontal cortex in adulthood [49], [63]. Similarly, in humans, it has been hypothesized that exposure to stressful events during childhood is associated with changes in neural function and disruptions of the HPA axis. It is these changes that could in turn lead to differences in health and achievement during adulthood [41].

The findings from the current study support this third hypothesis. Percent change in cortisol across the time of the fMRI scan was significantly related to both income-to-needs ratio and was related to both task performance and associated neural activation in the context of learning. These findings provide preliminary evidence in support of the theory that changes in stress reactivity are one way in which SES gets “under the skin” to affect child health and well-being.

The data presented here provide two possible accounts of the association between SES and prefrontal function in childhood. First, language-rich environments, such as those provided in many HSES homes, may affect the development of executive function by providing opportunities to “practice” components of such functioning at home. For instance, working memory is required for more complex language structures, such as conjunctions, to be understood. Increased use of complex sentences by parents means that children must more often hold in mind the beginning of a sentence, while waiting to understand the end of a sentence. Useful follow-up studies might identify natural experiments in which the complexity of the early language environment varies independently of SES and in which the effects of these environments on prefrontal function could be observed.

Second, exposure to adversity associated with SES may affect the development of the prefrontal cortex via a direct effect of exposure to stress hormones on neural development. This is consistent with previous work in rodents demonstrating that chronic exposure to glucocorticoids decreases dendritic spines in the medial prefrontal cortex [49]. Stress exposure as a pathway for the effect of SES is also consistent with current theories emphasizing the importance of protecting children from the stressful experiences that accompany low SES environments [47], [64].

This study had several limitations that should also be taken into account. Primary among them is its limited sample size, which can increase the likelihood of Type II errors and made it impossible to directly test the proposed pathways through a traditional mediation analysis. Thus all findings about the pathways by which SES comes to affect neural function and behavior must be taken as preliminary and hypothetical. Despite the relatively small number of participants, substantial and significant associations between SES and prefrontal function were observed. The strength of the observed association between SES and prefrontal function is not surprising given the profound influence that childhood SES has on child and adult outcomes. A second limitation is the inability to test a broad variety of environmental mediators that might plausibly link SES and executive function. Instead, we chose 3 hypothesis-driven aspects of childhood experience: language environment, child language use, and an index of the HPA axis function. Each of these exposures has a corresponding body of literature demonstrating associations with cognitive outcomes in childhood. In the current study, two of these predictors—the parental language environment and our index of HPA axis function—appeared significantly linked to both prefrontal function and task performance. The hypothesis-driven approach used here disallows broader exploration of the many variables possibly contributing to prefrontal development; however, it is more appropriate to the small sample size, and complexity of data collection, and analysis inherent in an fMRI study. Future studies should examine these potential mediators of the association between SES and executive function, in addition to other potential mediators, including structural barriers such as access to under-performing schools and increased exposure to adversities such as violence in the home and neighborhood. Finally, while we measure salivary cortisol in children, we do not examine the child or parent's self-report of stressful experiences. Directly assessing self-report of stress exposure may lead to stronger associations between cognitive or neural measures and stress.

Family SES in childhood is an important predictor of health and achievement in adulthood. Identifying the pathways by which SES comes to have such far-reaching influences can lead directly to possible policy interventions. Specifically, previous work has emphasized the importance of enrichment for children raised in low SES environments. If parental language environment had been the only variable associated with both family SES and child prefrontal function, this approach would continue to make sense. However, we observed a variable with these associations: percent change in salivary cortisol, an index of HPA axis function. These two observations, taken together, are consistent with the importance of addressing adversity associated with low SES in childhood in addition to advocating for enriching linguistic environments for all children.
